# Green synthesis of *Rhodobryum roseum*-mediated ZnO nanoparticles: a multifunctional evaluation of biomedical activities and agricultural applications

**DOI:** 10.1038/s41598-026-56052-0

**Published:** 2026-06-03

**Authors:** Wasim Akhtar, Kanwal Nisar, Sadaf Kayani, Iram Fatima, Tijen Demiral Sert, Farhat Gul, Abida Akbar, Asif Kamal, Sarah Abdul Razak, Raheek Alwaznah, Islem Abid, Muhammad Tahir Naseem

**Affiliations:** 1 Department of Botany, University of Azad Jammu and Kashmir, Muzaffarabad, Pakistan; 2 Department of Biology, Faculty of Engineering and Natural Sciences, Süleyman Demirel University, East Campus, Isparta, Türkiye; 3 Department of Botany, Mohi-ud-Din Islamic University, Nerian Sharif (Tararkhal) District Sudhnoti, Azad Jammu and Kashmir Pakistan; 4 Pakistan Council of Scientific and Industrial Research (PCSIR), Head Office, 1-Constitution Avenue, G-5/2, Islamabad, Pakistan; 5 Department of Plant Sciences, Faculty of Biological Sciences, Quaid-i-Azam University, Islamabad, 45320 Pakistan; 6 Department of Biotechnology, University of Azad Jammu and Kashmir, Muzaffarabad, Pakistan; 7 Institute of Biological Sciences, Faculty of Science, University of Malaya, 50603 Kuala Lumpur, Malaysia; 8 Centre of Excellence in Biotechnology Research (CEBR), DSR, King Saud University, P.O. 2455, Riyadh, 11495 Saudi Arabia; 9 Department of Electronic Engineering, Yeungnam University, Gyeongsan, 38541 South Korea

**Keywords:** Green synthesis, Bryophytes, ZnO-NPs, Antimicrobial, Impact on seed germination, Biochemistry, Biological techniques, Biotechnology, Microbiology, Nanoscience and technology, Plant sciences

## Abstract

Nanotechnology is a fast-growing field with diverse applications in various disciplines. Based on the applications in diverse fields and the medicinal significance, *R. roseum* was phytochemically investigated and was used for the synthesis of zinc oxide nanoparticles (ZnO-NPs). Biosynthesized NPs were characterized using UV spectrophotometry (UV), Fourier transform infrared spectroscopy (FTIR), X-ray diffraction (XRD), and scanning electron microscope (SEM), and used in antioxidant, antibacterial, and seed nano priming activities. Antibacterial activity was observed against *Klebsiella pneumoniae, Bacillus subtilis, Enterococcus faecalis,* and *Staphylococcus aureus,* with the maximum zone of inhibition recorded against *S. aureus* (23 mm ± 0.57) at 1000 µg/ml. ZnO-NPs exhibited the highest antioxidant activity (75.2%) at 100 µg/mL in comparison with the standard (ascorbic acid), which showed 79% activity at the same concentration. The IC50 value of *R. roseum*-mediated ZnO-NPs obtained was 35.79 at a 20 µg/mL ZnO-NP concentration. Seed priming with *R. roseum*-mediated ZnO NPs markedly enhanced maize growth, with the highest performance recorded at 200 ppm, yielding 3.33 ± 0.40 g fresh weight, 3.20 ± 0.1 g dry weight, and 6 ± 1 leaves. At this concentration, seedlings also exhibited increased root (13.5 ± 0.57 mm) and shoot length (12.6 ± 0.57 cm) along with elevated chlorophyll content (34.9 ± 4.57), indicating significant improvement in physiological attributes. Compared to the control, nanoparticle treatment at 50 µg/mL increased peroxidase activity from 18.4 to 31.3 U mg⁻^1^ protein, superoxide dismutase from 22.6 to 36.9 U mg⁻^1^ protein, and catalase from 15.2 to 27.8 U mg⁻^1^ protein. In conclusion, green-synthesized ZnO-NPs are effective and can be utilized in agriculture, biomedical, and other fields.

## Introduction

Nanotechnology is an emerging and rapidly advancing field that has revolutionized multiple scientific domains. It involves the design, fabrication, and application of nanostructures with dimensions typically ranging from 1 to 100 nm^1^. The unique properties of nanoparticles (NPs), including high surface area-to-volume ratio, reduced particle size, and tunable physicochemical characteristics, make them suitable for applications in biomedicine, agriculture, and environmental sciences. Various metals, such as gold (Au), silver (Ag), copper (Cu), zinc (Zn), selenium (Se), and iron (Fe), have been extensively utilized for the synthesis of nanoparticles^2^. Metallic nanoparticles include gold, silver, copper^3^, iron^4^, selenium^5^, and zinc-based systems. Among these, metal oxide nanoparticles such as ZnO, TiO_2_, and CuO have demonstrated significant antimicrobial potential^6,7^.

Zinc (Zn) is an essential micronutrient that plays a crucial role in plant metabolism, including antioxidant defense, membrane integrity, and stress tolerance^8^. Zinc oxide (ZnO), a crystalline inorganic compound^9^, has attracted significant attention due to its multifunctional biological and physicochemical properties^10^. ZnO-NPs exhibit diverse biomedical applications, including antibacterial, anticancer, anti-inflammatory, wound healing, and drug delivery functions^11^. These nanoparticles possess unique properties such as semiconductivity, piezoelectric behavior, optical transparency, chemical stability, low toxicity, and cost-effectiveness^12^, which enable their use in industrial and technological applications such as catalysis, coatings, pharmaceuticals, and optoelectronic devices^13^. Additionally, ZnO-NPs have been widely incorporated into antimicrobial formulations, including textiles, coatings, and packaging materials, to inhibit microbial growth^14^.

Recent advances in plant-mediated (green) synthesis of nanoparticles have gained considerable attention due to their eco-friendly, cost-effective, and sustainable nature. In particular, ZnO nanoparticles (ZnO-NPs) synthesized via biological routes have shown enhanced applications in antimicrobial, anticancer, and antioxidant systems compared to chemically synthesized counterparts^7^. For instance, doped ZnO-NPs (e.g., ZnO–SeO composites) have demonstrated broad-spectrum activity against bacterial and fungal pathogens, including *Staphylococcus aureus*, *Escherichia coli*, and *Candida albicans*, as well as cytotoxic effects against cancer cell lines such as MCF-7^15^. Despite these advances, the exploration of lower plant groups such as bryophytes for nanoparticle synthesis remains largely underexplored, highlighting a critical research gap.

Green synthesis of nanoparticles using plant extracts has emerged as a promising alternative to conventional chemical and physical methods, owing to its eco-friendly, cost-effective, and sustainable nature. This approach utilizes plant-derived phytochemicals such as phenolics, flavonoids, alkaloids, and proteins as natural reducing and stabilizing agents, thereby eliminating the need for toxic reagents^16,17^. Furthermore, plant-mediated synthesis not only minimizes environmental impact but also enhances the biological functionality of nanoparticles, making them highly suitable for agricultural and biomedical applications^18^. Recent studies have demonstrated that plant-derived nanoparticles can significantly improve antimicrobial efficacy and promote plant growth, reinforcing their potential in sustainable nanotechnology^19^. However, most existing studies have predominantly focused on higher plants, with limited exploration of bryophytes, which are known to possess unique and diverse phytochemical profiles. *Rhodobryum roseum* (Hedw.) Limpr. has rich phytochemical composition, including phenolics, flavonoids, amino acids, and fatty alcohols^20^, which can facilitate efficient nanoparticle reduction and stabilization. Moreover, previous reports have demonstrated that *R. roseum* exhibits significant antimicrobial, antioxidant, and anti-inflammatory activities^21,22^, further suggesting its potential to enhance the bioactivity of the synthesized nanoparticles.

The present study demonstrates a novel and integrative approach by employing the bryophyte *R. roseum* for the green synthesis of zinc oxide nanoparticles (ZnO-NPs), expanding the scope of plant-mediated nanotechnology beyond commonly studied higher plants. Unlike conventional methods, this work leverages the unique phytochemical profile of *R. roseum*, rich in phenolics, flavonoids, amino acids, and fatty alcohols, to facilitate efficient nanoparticle reduction and stabilization. The study not only provides detailed physicochemical characterization of the biosynthesized ZnO-NPs using UV–Vis, FTIR, XRD, and SEM but also evaluates their multifunctional biological activities, including potent antibacterial and antioxidant effects, as well as significant enhancement of maize growth through seed nano-priming. By integrating green synthesis with comprehensive biomedical and agricultural assessments, this research establishes *R. roseum* as a sustainable and innovative bryophyte-based bioresource for ZnO-NP production, offering promising applications in both agriculture and biomedicine.

## Materials and methods

### Plant collection and extract preparation

The plant material was collected from Kotli (33.5003° N, 73.9013° E), Azad Kashmir, during the summer season, identified by a qualified taxonomist (Amir Shehzad), and submitted to the AKASH Herbarium, having voucher no. AR-76865 at the Department of Botany, University of Azad Jammu and Kashmir. The Fresh leaves of *R. roseum* were washed, completely air-dried, and then powdered using an electric grinder. About 10 g of powder was added to 100 mL of distilled water and boiled at 80 °C for 10 min. The extract was filtered using Whatman filter paper and then stored for detailed analysis.

### Qualitative study on phytochemical constituents of phyto-extract

A qualitative phytochemical study includes the method of detecting and identifying different phytoactive components. It aims to assess the absence or occurrence of particular phytochemicals like flavonoids, phenolic compounds, alkaloids, protein, terpenoids, glycosides, saponins, oils, steroids, quinones, anthocyanins, and tannins. The Qualitative phytochemical assessment was achieved through the previous standard protocol^23^. This study provides essential information regarding the bioactive constituents of the plant, which can contribute to its therapeutic potential.

### Synthesis of *R. roseum* mediated ZnO-NPs

About 10 g of zinc sulphate hexahydrate salt (ZnSO_4_·6H_2_O) was dissolved in 200 mL of deionized water to prepare the precursor solution. The plant extract (100 mL) was then added to the zinc precursor solution in a 1:2 (v/v) ratio under continuous magnetic stirring at room temperature. The pH of the reaction mixture was adjusted and maintained at 7.4 to facilitate nanoparticle formation. Afterward, plant extract was added to the zinc solution till the solution started changing from colourless to pale brown, indicating the formation of suspended particles. The mixture was left overnight and filtered to obtain the suspended particles, which were dried in an oven at 80℃ for 4 h, then crushed into a very fine powder.

### Characterization of *R. roseum-mediated* ZnO-NPs

The absorption spectrum of the fabricated ZnO-NPs was determined using a UV–visible spectrophotometer (Shimadzu UV-1601). The size and morphology were accessed by JSEM-IT800. The functional groups of synthesized NPs were identified using FTIR (Alpha, Bruker, Germany)^24^. The nature of particles was determined by X-ray diffraction analysis (PANalytical Netherlands). The average crystallite size of ZnO-NPs was calculated using the Debye–Scherrer equation:

$$D = K\lambda /\left( {\beta \;\cos \theta } \right),$$

where *D* represents crystallite size, *K* is the shape factor (0.9), *λ* is the X-ray wavelength, *β* is the full width at half maximum (FWHM) of the diffraction peak (in radians), and *θ* is the Bragg angle.

### Antibacterial activity

The antibacterial activity of ZnO-NPs was evaluated against *Bacillus subtilis*, *Enterococcus faecalis, Staphylococcus aureus, Klebsiella pneumonia,* and *Escherichia coli* strains by the disc diffusion method utilizing a filter disc of 09 mm in diameter following the previous standard procedure^25^. ZnO-NPs (31, 62, 125, 250, 500, and 1000 µg /mL) were applied on these filter discs in petri plates and incubated for 24 h at 37 °C. Oxytetracycline was used as a control, and after 24 h, the zones of inhibition (ZOI) were measured with the help of a millimetre (mm) scale.

### Antioxidant activity

DPPH scavenging assay was performed using ascorbic acid as a standard. The synthesized NPs (20, 40, 60, 80, and 100 µg/mL concentrations) were mixed with the DPPH solution and then incubated at 25 ℃ for 40 min. The absorbance (517 nm) was measured, and the percentage inhibition was calculated^26^.

$$Percentage\,inhibition = \frac{(Absorbance\,control - Absorbance\,sample)}{Absorbance\,control} \times 100$$

### Seed priming with ZnO-NPs

Kashmir Gold Maize corn variety seeds were sterilized with 10% bleach solution, and then different dilutions of ZnO-NPs, *i.e.,* 50, 100, 200, 300, 400, and 500 ppm prepared in DMSO by suspending ZnONPs with an Ultrasonic machine (USC500W-23L80C). After making dilutions in Falcon tubes, the seeds were immersed and kept in the dark for 20 h. Subsequently, seeds were taken out from the falcons and air-dried for 1 day. Later, seeds were stored in the refrigerator at − 4 °C until planting**.** For the planting experiment, seeds were sown in pots containing a standardized soil mixture composed of loamy soil, sand, and organic compost in a 2:1:1 ratio. Each treatment consisted of three biological replicates with 10 seeds per replicate. Plants were grown under controlled environmental conditions with a 16/8 h light/dark photoperiod, temperature of 25 ± 2 °C, and relative humidity of 60–70%.

### Growth parameters

For each concentration, 10 seeds were taken and sown in pots. After planting, the seeds were observed daily, and then after 15 days, different growth parameters were measured, such as the root and shoot length, number of leaves, dry and fresh weight, and chlorophyll contents by using a SPAD meter. The length of root and shoot seedlings was measured with the help of a ruler, and then the fresh and dry weight of the seedlings was determined in grams.

### Effect on antioxidant system

Antioxidant enzyme activities, including peroxidase (POD), superoxide dismutase (SOD), and catalase (CAT), were assessed using standard spectrophotometric methods^27^. Untreated samples (without nanoparticle exposure) served as the control, while treated samples were exposed to different concentrations of biosynthesized nanoparticles. All assays were performed in triplicate, and enzyme activities were expressed as units per milligram of protein (mean ± SD).

### Statistics

All experiments were conducted in triplicate (n = 3), and the results are presented as mean ± standard deviation (SD) to ensure reliability and reproducibility. Statistical analyses were performed using GraphPad Prism 5. Differences among treatment groups were evaluated using one-way analysis of variance (ANOVA) followed by Tukey’s post hoc test to determine multiple comparisons. A *p*-value of less than 0.05 (*p* < 0.05) was considered statistically significant. For antioxidant assays, IC_50_ values were calculated using nonlinear regression analysis of dose–response curves. Antibacterial activity (zone of inhibition), seed germination parameters, growth attributes, and enzymatic antioxidant activities were statistically compared with the control group to assess the significance of nanoparticle treatment.

## Results and discussion

### Qualitative study of the phytochemicals

Qualitative evaluation of the extract was recognized by assessing phytochemical constituents that existed in the various segments of the plant, as depicted in Table 1. The qualitative study of *R. roseum* in distilled water extract was performed to identify the secondary metabolites such as flavonoids, terpenoids, alkaloids, quinones, glycosides, anthocyanins, phenolic compounds, saponins, oils, fats, tannins, and steroids. This complete profile is shown in Table 1. There is a minor difference in the Qualitative compounds of *R. roseum,* as reported by a previous researcher^28^, which may be due to the plant’s physiological responses to its environment. The presence of dominant phytochemicals, particularly phenolic compounds, flavonoids, and terpenoids, plays a crucial role in the green synthesis of nanoparticles^29^. These bioactive molecules act as natural reducing as well as stabilizing agents, facilitating the conversion of metal ions into nanoparticles while preventing their aggregation^30^. Moreover, their functional groups contribute to surface capping, thereby enhancing the stability, biocompatibility, and biological activity of the synthesized nanoparticles.

**Table 1:** Qualitative analysis of the extract of *R. roseum*.

Sr. no	Secondary metabolites	RRDW
1	Phenols	+ + +
2	Alkaloids	+ +
3	Flavonoids	+ +
4	Saponins	+ + +
5	Terpenoids	+ +
6	Tannins	+
7	Glycosides	+
8	Steroids	+
9	Anthocynins	+
10	Quinones	−
11	Protein	−
12	Oils and fats	+

+  +  +  = means to the copiousness, +  +  = confirm the modest amount, +  = shows to minute quantity, − = means to absence of phytoconstituents.

### UV–Vis analysis of ZnO-NPs

**Fig. 1:**
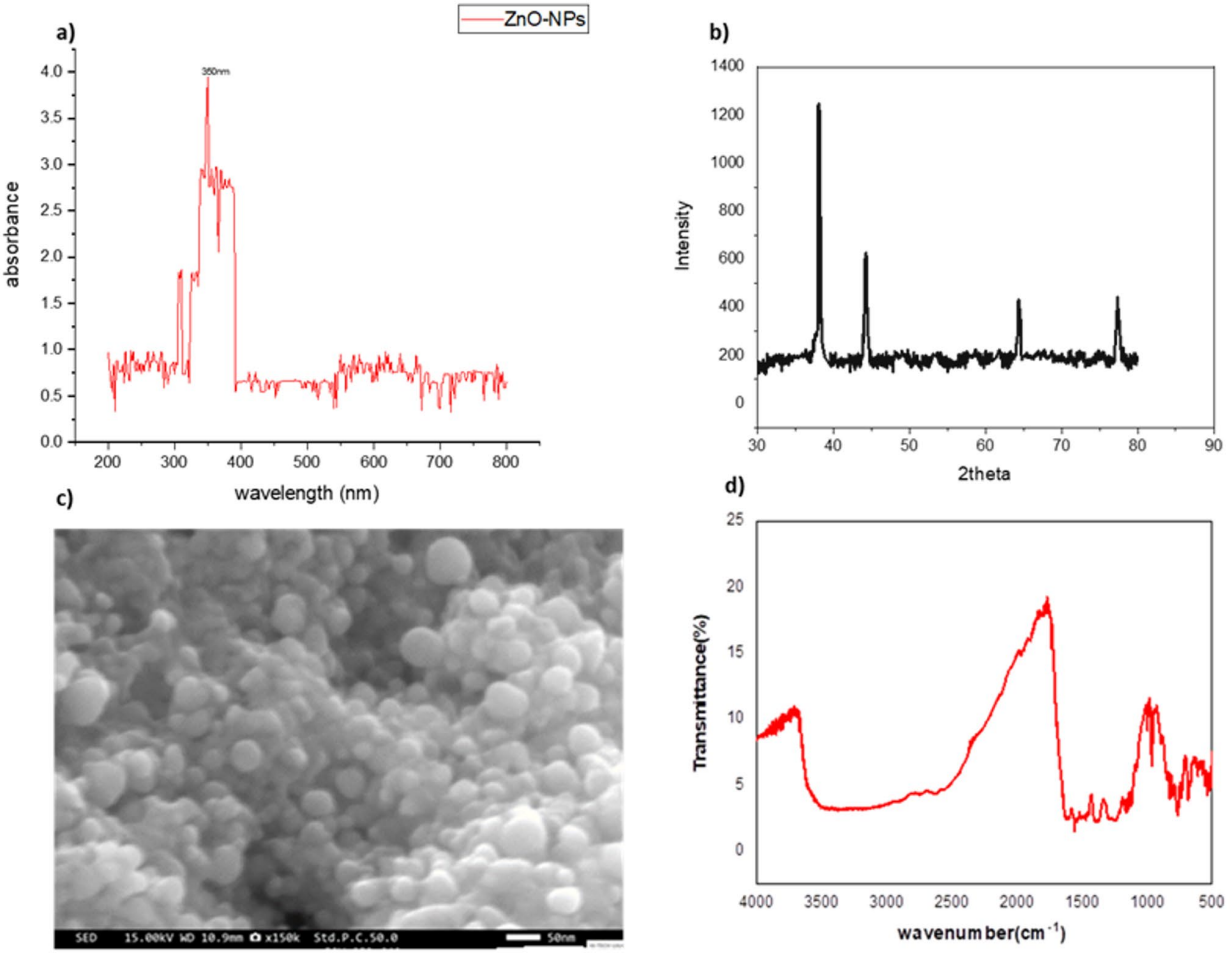
Characterization of ZnO-NPs using various techniques (**a**) UV visible spectra (**b**) XRD pattern, (**c**) SEM analysis, (**d**) FTIR analysis.

During nanoparticles synthesis change in solution colour is due to the change of metal ions from a higher to a lower oxidation state. UV–Vis spectrophotometer analysis of the reaction media further supported the Zn^+2^ ions to Zn. UV–Visible spectroscopy spectral analysis demonstrated strong absorption bands between 350 nm, which confirmed the synthesis of ZnO-NPs in the reaction mixture. Our study also correlates with the earlier reports^31^, indicating the maximum absorbance of *R. roseum* mediated ZnO-NPs at 350 nm (Fig. 1a).

### X-ray diffraction

To confirm the biosynthesis, the crystallographic structure of ZnO-NPs was assessed using XRD. The air-dried powder of ZnO-NPs was used for XRD. For this, XRD had shown remarkable peaks at 2θ of 36.06, 38.47, 40.67, and 48.46, which were due to crystal planes such as 111, 200, 220, and 311 of the crystal system, representing the face-centered cubic system of ZnO-NPs (Fig. 1b). The average size of the nanoparticle was calculated to be 47 nm. Previously, Naveed et al.^31^ also indicated the face-centered cubic crystalline structure of biosynthesized ZnO-NPs, which has been observed in our study. Moreover, the formation of different peaks showed that the NPs containing organic compounds play a key role in the reduction of zinc ions and stabilization of ZnO-NPs.

### SEM analysis

The surface morphology of fabricated ZnO-NPs was investigated using JSEM, as presented in Fig. 1c. The SEM images of biogenically synthesized ZnO-NPs showed that ZnO-NPs were spherical in shape (Fig. 1c). In addition to shape, the SEM micrographs indicate a relatively heterogeneous particle size distribution, with nanoparticles appearing in both isolated and clustered forms. This variation suggests partial aggregation, which is commonly observed in biogenic nanoparticle synthesis due to the presence of phytochemicals acting as capping and stabilizing agents. The observed aggregation may be attributed to interparticle interactions such as van der Waals forces and hydrogen bonding, leading to the formation of nanoparticle clusters. However, such aggregation does not necessarily diminish functionality; rather, it may enhance certain biological activities by facilitating localized concentration effects. These morphological characteristics may play a crucial role in the enhanced antibacterial, antioxidant, and seed nano-priming activities observed in this study^32^. The NPs with a smaller size have a larger surface area and stronger biological activities.

Furthermore, the SEM observations are consistent with the particle size trends inferred from XRD analysis, where nanoscale crystallite size supports the formation of fine ZnO particles. The slight discrepancy between individual particle size and aggregated clusters observed in SEM is expected, as SEM reflects the surface morphology and agglomeration state rather than primary crystallite size. The NPs with a smaller size have a larger surface area and stronger biological activities. Thus, *R. roseum*-mediated ZnO-NPs have revealed stronger antibacterial and antioxidant properties. These results are aligned with previous researchers^33,34^.

### Fourier-transform infrared spectroscopy

The functional groups responsible for the chemical bonding and inner mechanisms of formation were determined by FTIR. The functional group area and the fingerprint region are the two primary regions that make up the FT-IR spectrum. The formation of FTIR peaks was observed at 3450, 1587, 1407, 1260, 1150, 1032, 915, 760, 650, and 450–500 cm^−1^. For phenol, the band at 3450 cm^-1^ indicated the existence of –OH stretching as previously^35^. The formation of a peak at 1587 cm^−1^ represented the presence of 1° amines, and this is consistent with the previous studies^36^. The formation of strong peaks at 1407, 1260, 1150, 1032, 915, 760, and 650 cm^−1^ indicated the carboxyl groups in the amino acid C–H wagging of alkyl halides, C–O–C symmetric stretching, –CH = CH_2_ aromatic compound, C–N and C–Cl stretching of alkyl halides and halogens (Cl–Br) (Fig. 1d). Earlier reports^37^ also showed the presence of functional groups in NPs that are involved in the synthesis and stabilization of NPs.

An interpretation of these FTIR results indicates that specific biomolecules present in the extract play distinct roles during nanoparticle synthesis and stabilization. Phenolic compounds and flavonoids, due to their hydroxyl groups, primarily act as reducing agents, facilitating the conversion of Zn^2^⁺ ions into ZnO nanoparticles^38^. Simultaneously, proteins, amino acids, and polysaccharides contribute to stabilization by binding to the nanoparticle surface through functional groups such as –OH, –NH, and –COOH, forming a capping layer that prevents aggregation. This correlation between FTIR-identified functional groups and phytochemicals clearly demonstrates that the biogenic synthesis and stabilization of nanoparticles are governed by the synergistic action of these plant-derived biomolecules.

### Antibacterial activity

**Fig. 2:**
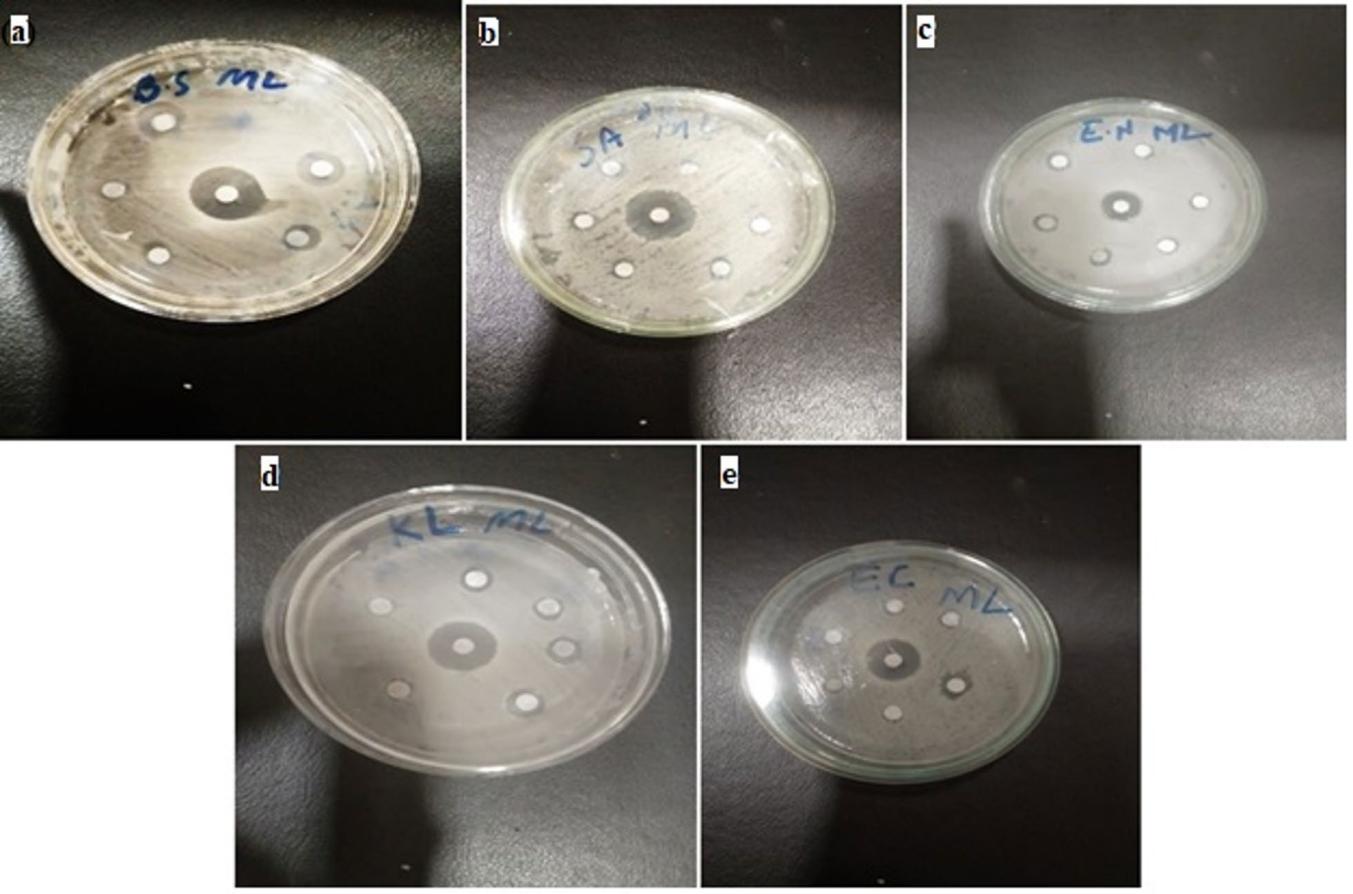
Antimicrobial activity of ZnONPs observed against various strains (**a**) *Bacillus subtilis*, (**b**) *Enterococcus faecalis*, (**c**) *Staphylococcus aureus*, (**d**) *Klebsiella pneumonia* and (**e**) *Escherichia coli.*

**Fig. 3:**
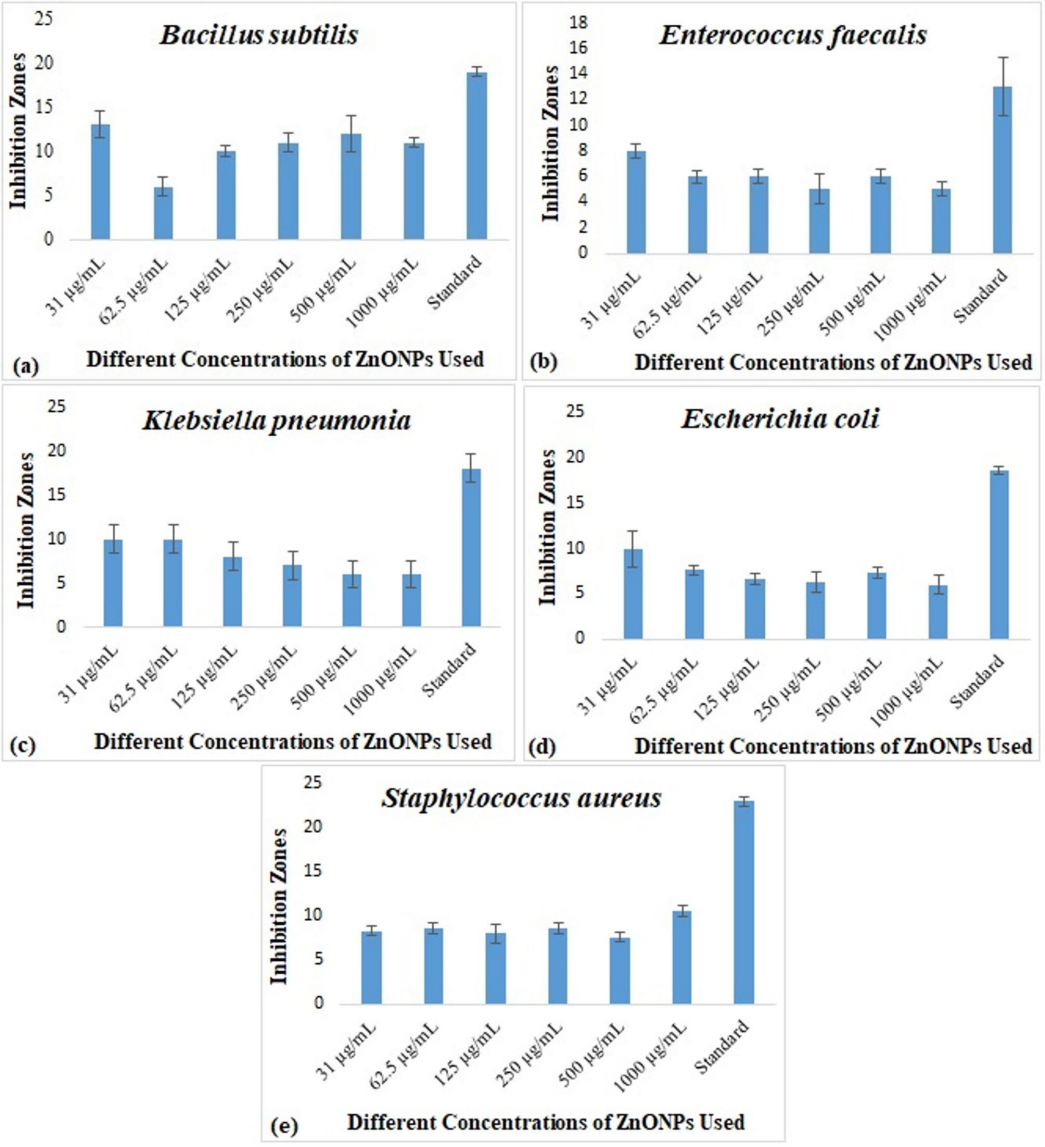
Zones of inhibition recorded in *R. roseum* against various bacterial strains (**a**) *Bacillus subtilis* (**b**) *Enterococcus faecalis* (**c**) *Staphylococcus aureus*, (**d**) *Klebsiella pneumonia*, (**e**) *Escherichia coli.* The data indicate mean ± SD, and oxytetracycline was used as a standard.

The antibacterial efficacy of *R. roseum*-mediated ZnO-NPs was investigated against five pathogenic bacteria. The biogenically synthesized ZnO-NPs have shown maximum bactericidal ability towards all five pathogenic bacteria. The results showed a maximum bacteriostatic effect against *S. aureus*, with a diameter of 23 mm ± 0.57 at a concentration of 1000 µg/mL, followed by *B. subtilis*, with a diameter of 19 mm ± 0.577 at a concentration of 1000 µg/mL. *K. pneumonia* and* E. coli* showed antibacterial activity with 18 mm ± 0.57 and 18 ± 1 zone of inhibition at 1000 µg/mL, and the minimum antibacterial effect was shown against *Enterococcus faecalis,* which was 13 mm ± 0.57 at 1000 µg/mL (Figs. 2 and 3). These findings are compared with the previous study, which observed that at higher concentrations of ZnO-NPs, the ZOI also increased^39^. Similarly, it has been reported maximum anti-microbial potential of *R. roseum* in ethanol and acetone extracts against *Sclerotium rolfsii* has a 5.00 µg/mL MIC value^18^. Moreover, myco-derived ZnO-NP exhibited the antifungal potential against *Fusarium* *nygamai* causative agent of dry rot in potato, at 100 µg/mL with a 21 ± 0.4 mm reduction in mycelial growth as compared with the control^40^. The antibacterial mechanism of NPs is mainly attributed to the generation of reactive oxygen species (ROS), including hydroxyl radicals and superoxide ions, which induce oxidative stress and damage bacterial cell components^41^. Additionally, ZnO-NPs can disrupt cell membrane integrity, increase membrane permeability, and release Zn^2+^ ions, which interfere with intracellular metabolic processes, ultimately leading to bacterial cell death.

However, some studies suggest that bimetallic and nanocomposites are more effective in antimicrobial and antifungal applications. Previously, it has been reported minimum inhibitory concentration (MIC) was 25 µg /mL against *S. aureus* and *B. subtilis,* while 50 and 100 µg/mL demonstrated MIC against *K. pneumonia* and *E. coli* strains^41^. Further, Zn-Au bimetallic NPs also showed potential inhibition against some fungal strains. The synergism of combined metals can accelerate antimicrobial and multispectral potential^42^. Such results were also supported by Zn-Mn nanocomposites, and they’re in silico molecular docking against some bacterial strains^43^.

### Antioxidant activity

**Fig. 4:**
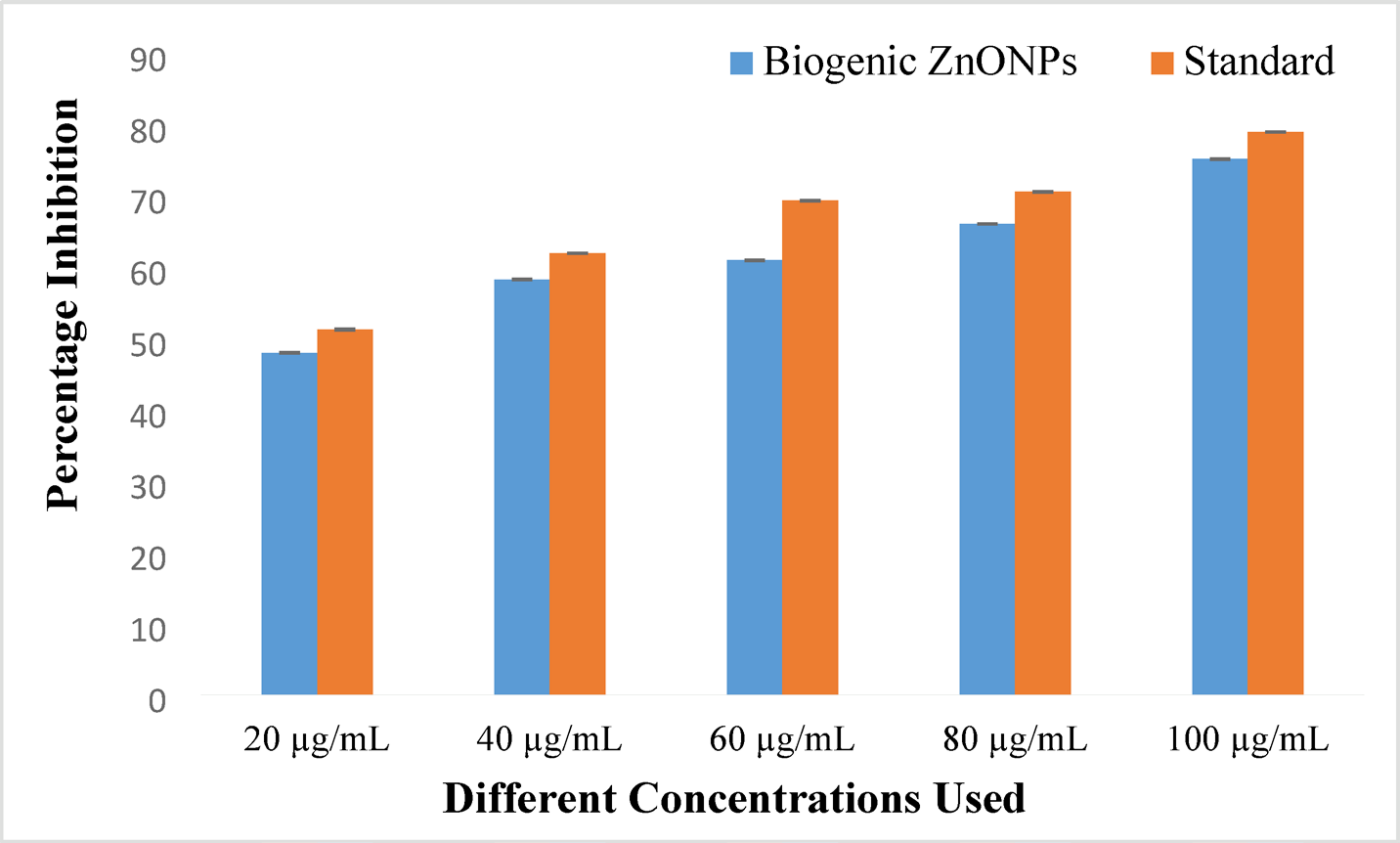
The antioxidant activity observed in biosynthesized ZnO-NPs of *R.roseum,* along with the standard Ascorbic acid. Data indicate mean ± SD, and ascorbic acid was used as a standard.

Results from the DPPH (2,2-Diphenyl-1-picrylhydrazyl) method were interpreted using the IC_50_ parameter. It measures the antioxidant potential by monitoring the decrease in purple DPPH radical to a yellow (or colourless) form when mixed with a sample. It typically utilizes a spectrophotometer at 517 nm after 30 min of incubation in the dark. A DPPH solution of 4 mg dissolved in 100 mL of methanol was made, and it was left in the dark for half an hour. Five distinct doses of biosynthesized ZnO-NPs, namely 20 μg/mL, 40 μg/mL, 60 μg/mL, 80 μg/mL, and 100 μg/mL, were prepared and utilized. In test tubes, these sample concentrations were combined with the DPPH solution. Following that, test tubes were incubated for 40 min at 25 °C. The lower IC_50_ value represents the higher scavenging activity. In the present work, the highest antioxidant activity of 75.2% was recorded at 100 µg/mL, while the standard (ascorbic acid) showed 79% at this concentration (Fig. 4). The IC_50_ value of *R. roseum* mediated ZnO-NPs obtained was 35.79 ppm, and the standard showed a 18.23 ppm value. It has been reported that *R. roseum* leaf extracts exhibit significant antioxidant potential and cardio-protective activity^22^. Our study also correlates with the earlier reports that indicated significant antioxidant activity in *R. roseum* methanol extract^44^. Importantly, the observed antioxidant activity can be mechanistically attributed to the surface-bound phytochemicals on ZnO-NPs, particularly phenolics and flavonoids, which facilitate electron transfer (ET) and hydrogen atom transfer (HAT) mechanisms to neutralize DPPH radicals^45^. These biomolecules donate electrons or hydrogen atoms to stabilize the free radicals, converting them into non-reactive species. Additionally, the nanoscale size and high surface area of ZnO-NPs enhance the availability of active sites, thereby improving interaction with DPPH radicals and increasing scavenging efficiency. The presence of functional groups such as –OH and –COOH on the nanoparticle surface further supports redox reactions and radical quenching.

When compared with previously reported plant-mediated ZnO nanoparticle systems, the IC_50_ value obtained in this study (35.79 ppm) indicates competitive antioxidant performance^46^. For instance, ZnO-NPs synthesized using *Camellia sinensis*^47^ and *Azadirachta indica*^48^ extracts have shown IC_50_ values typically ranging between 30 and 60 ppm, while other plant-based ZnO-NPs have reported values up to 70 ppm depending on synthesis conditions and phytochemical composition. Although the IC_50_ of the present ZnO-NPs is higher than that of ascorbic acid, it demonstrates strong antioxidant potential compared to many reported green-synthesized ZnO systems, highlighting the effective role of *R. roseum* phytochemicals in enhancing nanoparticle bioactivity. Bimetal NPs can exert more antioxidant capacity than single metal base NPs, as revealed in Zn-CU NPs, and others due to their combined and increased electron transfer, as well as neutralization of ROS through H atom transfer and single atom transfer^49,50^.

### Effects on seed germination

**Fig. 5:**
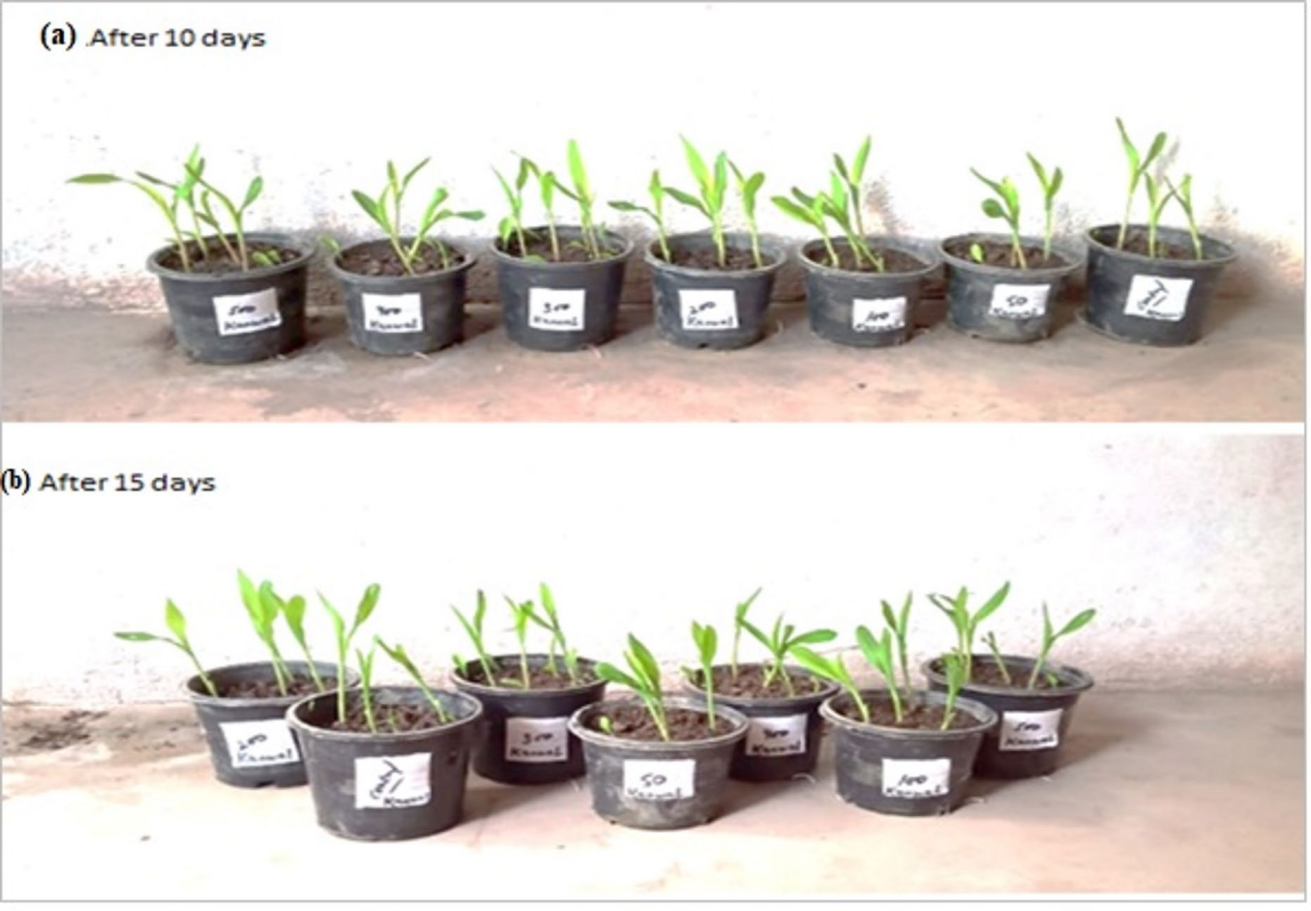
Growth of nano-primed Kashmir Gold Maize seedlings (**a**) After 10 days (**b**) After 15 days.

**Fig. 6:**
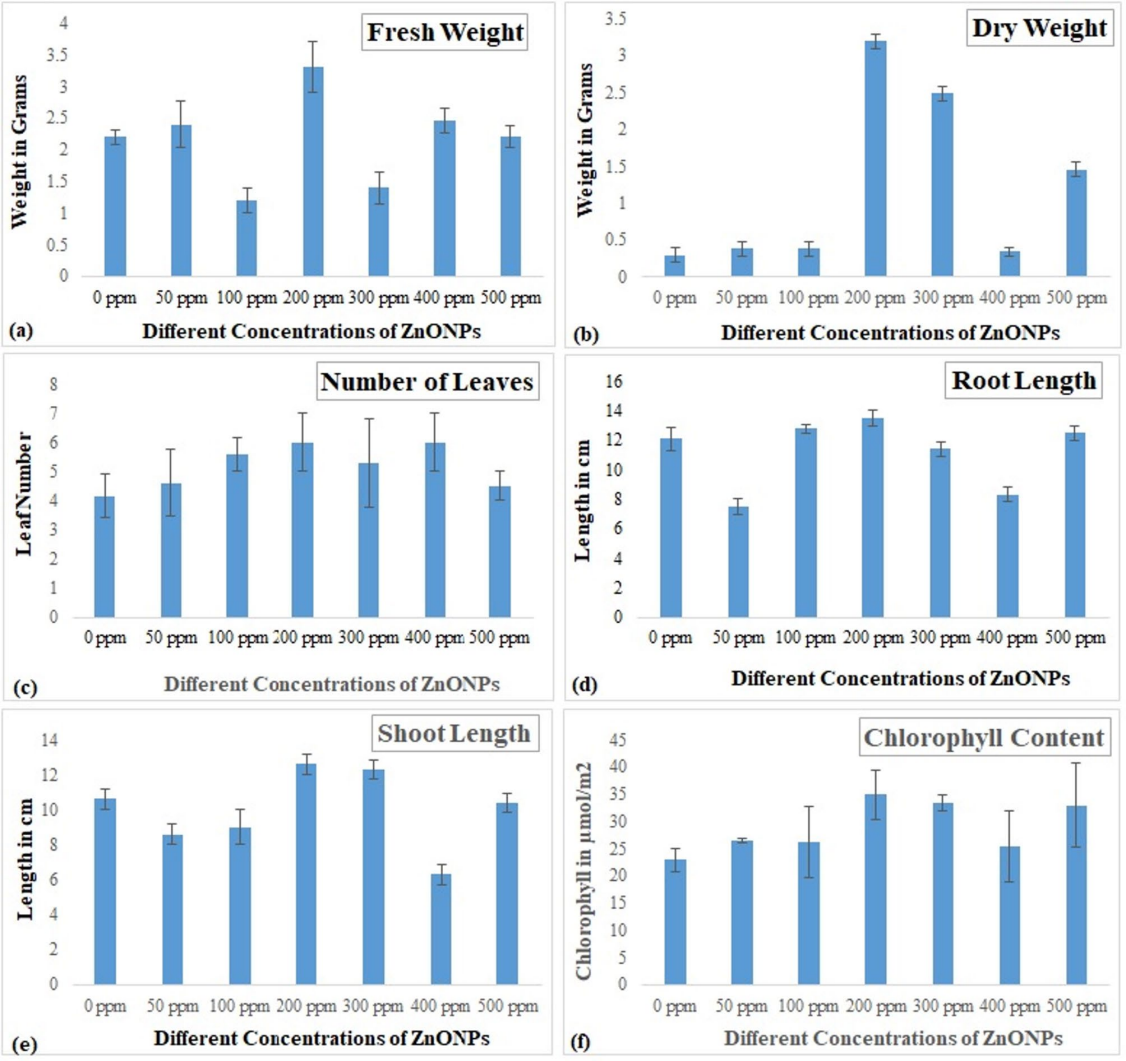
Graph representing the effect of different concentrations of green synthesized *R. roseum-* ZnO-NPs on Kashmir gold maize seed germination (**a**) Fresh Weight (**b**) Dry Weight (**c**) Number of Leaves (**d**) Root Length (**e**) Shoot Length (**f**) Chlorophyll Content.

Seed priming with ZnO NPs increases the zinc content in seeds and enhances the seedling growth^51^. ZnO is not easily soluble in soil; however, ZnO-NPs are chemically active and provide easy access of zinc to the plants^52^. The ZnO-NPs also elevate the concentration of photosynthetic pigments and the number of light reaction centres, which ultimately improves the photosynthetic performance^53^. In the current study, the synthesized NPs were applied to maize seedlings to enhance their growth and germination. First germination was observed at 300 ppm concentration after 4 days, and the maximum growth was recorded at 200 ppm. At low concentrations, the crop showed reduced growth, while the maximum growth rate was observed at the highest (200 ppm) concentration. At 200 ppm, 3.33 ± 0.40 g fresh weight, 3.20 ± 0.1 g dry weight, and 6 ± 1 leaves were observed. Similarly, root and shoot length were found to be 13.5 ± 0.57 mm and 12.6 ± 0.57 cm, and chlorophyll content was revealed as 34.9 ± 4.57 at 200 ppm concentration, as shown in Figs. 5 and 6. Many researchers^54,55^ have reported the positive effects of plant-based NPs on the growth of crops; however, the effect of *R. roseum*-mediated ZnO-NPs on the germination of maize seedlings has been investigated for the first time. Recent reports about ZnONPs primed seeds showed 100% germination along with improved vigor indices that can be attributed to high surface area, which ultimately increases seed interaction, water uptake, metabolic and enzymatic activation^51,56^.

Mechanistically, the enhanced growth can be attributed to the role of Zn as an essential micronutrient involved in enzyme activation, protein synthesis, and the regulation of plant growth hormones such as auxins^57^. ZnO-NPs facilitate improved water uptake during seed imbibition due to their large surface area, which accelerates metabolic activation and early germination processes^58^. Furthermore, ZnO-NPs may stimulate antioxidant defense systems in plants, reducing oxidative stress by scavenging reactive oxygen species (ROS), thereby promoting healthier seedling development^59^. The increased chlorophyll content observed at 200 ppm also suggests improved chloroplast development and photosynthetic activity, likely due to Zn-mediated activation of enzymes involved in chlorophyll biosynthesis.

In comparison with previous studies, plant-mediated ZnO-NPs have shown similar growth-promoting effects across different crops. Like, ZnO-NPs synthesized using *Azadirachta indica*^60^ and *Moringa oleifera*^61^ extracts have been reported to significantly enhance germination rates, biomass accumulation, and chlorophyll content in crops such as wheat and maize. Reported optimal concentrations generally range between 100 and 300 ppm, which aligns well with the findings of the present study, where 200 ppm yielded maximum growth. Additionally, earlier reports have demonstrated up to 100% germination and improved vigor indices in ZnO-NP-treated seeds, attributed to enhanced enzymatic activity, nutrient mobilization, and increased seed–nanoparticle interaction due to high surface area^62^. The present findings are therefore consistent with and extend these observations by demonstrating similar effects using *R. roseum*-mediated ZnO-NPs.

These results highlight the potential of green-synthesized ZnO-NPs in sustainable agricultural nanotechnology. Their ability to enhance seed germination, nutrient uptake, and photosynthetic performance suggests their utility as nano-enabled fertilizers or seed priming agents. Moreover, the use of plant-mediated synthesis ensures eco-friendliness, reduced toxicity, and improved compatibility with biological systems. This study provides a foundation for future research on dose optimization, field-scale applications, and long-term environmental impacts, paving the way for the integration of nanotechnology into modern agricultural practices.

### Effect on the antioxidant enzymes

The activities of antioxidant enzymes, including peroxidase (POD), superoxide dismutase (SOD), and catalase (CAT), were significantly enhanced in samples treated with biosynthesized nanoparticles compared to the control (Table 2). A dose-dependent increase in enzyme activities was observed up to 50 µg/mL nanoparticle concentration. At this concentration, POD, SOD, and CAT activities increased by approximately 70%, 63%, and 83%, respectively, relative to the control. However, a slight decline in enzyme activities was recorded at the highest nanoparticle concentration (100 µg/mL), though the values remained higher than those of the untreated control.

**Table 2:** Effect of biosynthesized nanoparticles on antioxidant enzyme activities. POD, SOD, and CAT activities increased dose-dependently up to 50 µg/mL, with slight decline at 100 µg/mL, remaining higher than control. This indicates enhanced antioxidant defense under nanoparticle treatment.

Treatment	Peroxidase (POD) (U mg^−1^ protein)	Superoxide Dismutase (SOD) (U mg^−1^ protein)	Catalase (CAT) (U mg^−1^ protein)
Control	14.4 ± 0.9	16.6 ± 1.1	12.2 ± 0.8
NP (25 µg/mL)	24.7 ± 1.2	29.8 ± 1.3	21.5 ± 1.0
NP (50 µg/mL)	31.3 ± 1.5	36.9 ± 1.6	27.8 ± 1.2
NP (100 µg/mL)	28.6 ± 1.4	33.2 ± 1.4	± 1.1

Antioxidant enzymes play a crucial role in mitigating oxidative stress by scavenging reactive oxygen species (ROS) generated under biotic and abiotic stress conditions^62^. In the present study, nanoparticle treatment markedly enhanced the activities of POD, SOD, and CAT, indicating an improved antioxidant defense mechanism. The increased SOD activity suggests efficient dismutation of superoxide radicals into hydrogen peroxide, while elevated CAT and POD activities contribute to the detoxification of hydrogen peroxide into water and oxygen. The observed dose-dependent enhancement up to an optimal concentration (50 µg/mL) may be attributed to the stimulatory effect of nanoparticles on cellular redox homeostasis and enzyme activation. Similar trends have been reported in previous studies, where biosynthesized nanoparticles acted as elicitors, inducing antioxidant enzyme responses^63,64^. The slight reduction at higher concentration could be due to nanoparticle-induced oxidative stress or enzyme saturation effects, a phenomenon commonly associated with excessive nanoparticle exposure^65,66^. Overall, the results demonstrate that biosynthesized nanoparticles positively modulate antioxidant enzyme activities at appropriate concentrations, thereby enhancing the oxidative stress tolerance potential of the system under study.

## Conclusion

In this study, green synthesis of zinc oxide nanoparticles (ZnO-NPs) using *R. roseum* leaves was successfully achieved, yielding spherical nanoparticles of 25–50 nm. Comprehensive characterization through UV–Vis, FTIR, XRD, and SEM confirmed their crystalline structure, functional groups, and morphology. The biosynthesized ZnO-NPs exhibited strong antibacterial activity against Gram-positive and Gram-negative bacteria, notable antioxidant potential (IC50 = 35.79 µg/mL), and enhanced antioxidant enzyme activities (POD, SOD, CAT) in treated seedlings. Seed nano-priming with these nanoparticles significantly improved maize growth, including root and shoot development, fresh and dry biomass, leaf number, and chlorophyll content, with optimal effects observed at 200 ppm. While these findings highlight the multifunctional potential of *R. roseum*-mediated ZnO-NPs for agriculture and biomedical applications, the study is limited to controlled laboratory conditions. Future research should focus on field-level validation, mechanistic insights into nanoparticle–plant interactions, and evaluation of environmental safety and biocompatibility. Overall, these results provide a strong foundation for developing sustainable nanoparticle-based technologies.

## Data Availability

Data will be available on request from the corresponding author.
